# Impact of Exercise Modalities on Upper Extremity Spasticity in an Adult with Quadriplegic Cerebral Palsy: A Case Report

**DOI:** 10.3390/jfmk10020177

**Published:** 2025-05-15

**Authors:** Juntack Oh, Michele Aquino

**Affiliations:** Department of Health and Sport Sciences, Adelphi University, Garden City, NY 11530, USA; maquino@adelphi.edu

**Keywords:** spasticity, cerebral palsy, exercise, physical activity

## Abstract

**Background:** Spasticity, a hallmark of quadriplegic cerebral palsy (CP), severely impacts mobility and quality of life. While exercise is known to enhance fitness and motor function in individuals with CP, its specific efficacy in reducing upper extremity spasticity remains insufficiently studied. This research investigated the effects of weight-resistance exercise (RE), hand cycle bike exercise (BE), and aquatic exercise (AE) on upper extremity spasticity in an adult with quadriplegic CP. **Method:** The participant was a 35-year-old individual with quadriplegic spastic CP, presenting severe spasticity in the right upper extremity and lower limbs, and milder left arm involvement. Dependent on a power wheelchair, they were cognitively intact, college-educated, and had participated in a community exercise program for five years. Over nine weeks, the participant completed 18 sessions—6 per modality of RE, BE, and AE—with each session held twice weekly for 50 min. Spasticity was assessed using the Modified Ashworth Scale (MAS) before and after sessions, with comprehensive pre- and post-intervention evaluations. **Result:** Total MAS scores decreased significantly from 2.76 to 2.33 (*p* < 0.05). AE yielded the largest reduction (2.81 to 2.10), followed by BE (2.75 to 2.36) and RE (2.72 to 2.54). ANOVA confirmed AE’s superior efficacy (F(2,15) = 27.20, *p* < 0.001, ηp^2^ = 0.78), with a 0.33 reduction overall. **Conclusions:** AE was most effective, likely due to buoyancy, followed by BE, with RE showing the least impact. These findings highlight aquatic interventions as promising for spasticity management in CP, necessitating further longitudinal, multi-participant research.

## 1. Introduction

Cerebral palsy (CP) encompasses a broad spectrum of motor disorders resulting from damage to the developing brain, leading to limitations in both gross and fine motor skills. CP is commonly classified into distinct motor types based on the dominant movement abnormality: ataxia, dyskinesia, or spasticity. Ataxic CP primarily affects coordination and balance, often resulting in unsteady, shaky movements. Dyskinetic CP is characterized by involuntary, uncontrolled movements stemming from fluctuating muscle tone [1]. Spasticity, the most prevalent feature of CP, is characterized by increased muscle tone and stiffness that significantly hinders smooth, efficient movement and functional mobility. In individuals with spastic CP, this neuromuscular impairment can lead to difficulties with ambulation due to excessive flexor tone in the lower limbs, as well as challenges with reaching and grasping tasks resulting from increased tone in the upper extremities [2]. This heightened muscle tone often causes pain, which is further exacerbated by muscle spasms, joint deformities, and postural abnormalities, all of which negatively impact mobility, sleep, and emotional well-being [3]. Addressing spasticity and pain is critical in enhancing functional independence and improving the overall quality of life for individuals with CP, particularly those with more severe forms like quadriplegic CP [4].

Quadriplegic CP, characterized by spasticity affecting all four limbs, poses additional challenges due to the significant impairment of both upper and lower extremities. Nearly half of all individuals with CP experience impaired upper extremity function due to spasticity, leading to reduced muscle strength and limited range of motion. In individuals with quadriplegic CP, this impairment is even more pronounced, often resulting in severe functional limitations in daily activities [5]. Additionally, those with CP are often at a heightened risk of developing metabolic and cardiovascular conditions due to their tendency toward a sedentary lifestyle and lower levels of health-related fitness [5]. Therefore, addressing spasticity in both the upper and lower extremities is essential not only for improving motor function, but also for mitigating associated health risks and promoting overall well-being.

Physical activity has emerged as a critical intervention in managing spasticity and pain for individuals diagnosed with CP, especially in cases of quadriplegia. Tailored exercise programs offer a comprehensive approach to addressing these challenges [6]. Engaging in physical activity tailored to their abilities allows individuals with quadriplegic CP to benefit from reduced muscle tone, increased flexibility, and improved muscle strength and endurance [3,7,8]. Specifically, physical activity and muscle strengthening training can help manage spasticity through repeated neuromuscular engagement, while strength training enhances functional capacity, supporting greater independence in activities of daily living [9]. Furthermore, physical activity has been positively linked to enhanced social and physical quality of life and increased happiness [10,11]. Incorporating physical activity into daily routines not only improves mobility and decreases pain, but also has a profound impact on psychosocial well-being, fostering a greater sense of fulfillment and joy for individuals with CP, particularly those with quadriplegia [12].

A growing body of literature has examined the benefits of various exercise interventions for individuals with CP. Recent reviews indicate that a wide range of exercises—including multi-joint resistance training, treadmill walking, stationary cycling, and aquatic exercise—have been shown to improve fitness levels and motor function [7,13,14]. Commonly used interventions include resistance training, arm ergometer exercises, and aquatic therapy, with primary outcomes focusing on muscular strength, cardiorespiratory fitness, and gait function [14,15,16,17,18,19,20]. These studies highlight the importance of incorporating diverse exercise modalities to meet the unique needs of individuals with CP, particularly those with quadriplegic involvement, to optimize physical function and overall health.

However, while many studies have explored spasticity as a secondary outcome, its specific improvement is often underemphasized. The evidence supporting the positive effects of exercise on spasticity remains limited, with only a few studies focusing on this area. Some research has suggested that resistance training can improve muscle quality in individuals with CP without worsening spasticity [15,16]. While significant reductions in spasticity may not always be observed, maintaining the current level of spasticity is crucial, as unmanaged spasticity can progressively worsen over time [21]. Therefore, while the direct impact of exercise on reducing spasticity may not always be pronounced, its role in preventing further deterioration and preserving function is an important consideration, especially for individuals with quadriplegic CP.

In addition to resistance training, other forms of exercise, such as cycling and arm ergometer exercises, have shown potential in enhancing cardiorespiratory fitness for individuals with neuromuscular conditions, including quadriplegic CP [22]. Both stationary and dynamic cycling options can be adapted to meet the needs of individuals with limited mobility [17,20]. Beyond improving cardiorespiratory fitness, these exercises may help maintain joint range of motion, which is crucial for preventing contractures and mitigating spasticity [17,20]. This dual impact on fitness and mobility underscores the therapeutic potential of cycling and arm ergometer exercises, making them valuable components of rehabilitation for individuals with quadriplegic CP.

Aquatic exercise is another highly recommended intervention for individuals with CP, including those with quadriplegia [14]. The buoyancy provided by water allows individuals to experience freedom of movement that may be difficult on land. Activities such as jumping, hopping, and running can be performed with minimal joint impact, fostering a sense of mobility and independence [23]. Moreover, water resistance naturally enhances muscle strength and cardiorespiratory fitness, while also providing a soothing environment that can reduce spasticity and improve overall flexibility [24]. The unique properties of water create a supportive environment where individuals with quadriplegic CP can engage in therapeutic activities that promote physical and psychosocial well-being.

Although the existing literature underscores the benefits of various exercise modalities, findings on their impact on spasticity, particularly in the upper extremities, remain inconclusive. Further research is needed to specifically address spasticity in this area [23,25,26]. To address this gap, this study aimed to investigate the effects of three distinct exercise interventions—resistance training, hand cycling, and aquatic exercise—on spasticity in the upper extremities of an adult diagnosed with quadriplegic CP. Through this research, we hope to contribute to the development of more effective therapeutic interventions tailored to the unique challenges faced by individuals with quadriplegic CP, with a focus on improving spasticity and overall quality of life.

## 2. Materials and Methods

### 2.1. Participant

The participant in this study was a 35-year-old male diagnosed with quadriplegic spastic CP, a severe form of CP that affects all four limbs and is characterized by significant muscle tone abnormalities and motor impairments. He experienced pronounced spasticity in the upper right extremity and both legs, resulting in reduced range of motion and difficulties with voluntary movement and coordination. The left arm showed comparatively less spasticity but was still affected by the overall condition.

Due to the severity of his spasticity, the participant used a power wheelchair for mobility, as he was unable to walk or use a manual wheelchair effectively. Despite these challenges, he had no cognitive impairments and had completed a college education, demonstrating intellectual competence. His commitment to managing his condition was evident in his active participation in a community-based exercise program over the past five years, which included activities like aquatics, boccia, resistance exercise, and hand cycling.

The participant formed a strong rapport with the researcher and expressed interest in exploring different exercise modalities to further manage his spasticity. This study was approved by the University Institutional Review Board, and the participant provided informed consent after being fully briefed on the study’s objectives, risks, and benefits, ensuring his voluntary participation.

### 2.2. Procedure and Intervention

**Figure 1:**
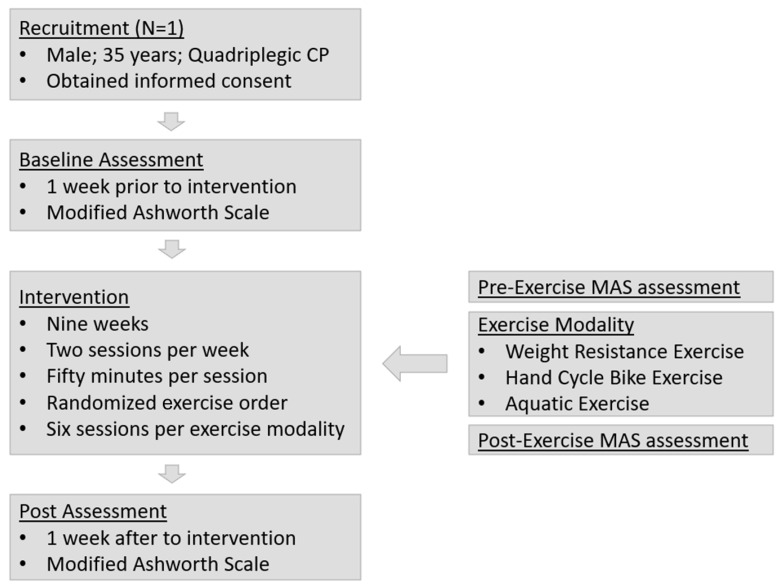
Procedure and timeline of the intervention.

The participant participated in a structured exercise program involving three modalities: weight-resistance exercise (RE), hand cycle bike exercise (BE), and aquatic exercise (AE). The intervention consisted of two sessions per week, each lasting 50 min, for a total of 18 sessions over 9 weeks. Each session featured a different exercise modality, ensuring that all three modalities were performed six times. To control for biases, the order of the modalities was randomized, with no modality occurring in consecutive sessions. This approach minimized carryover effects and allowed for a clearer assessment of the specific impact of each exercise modality on spasticity. Each modality was designed to engage the muscles similarly in terms of intensity and muscle involvement. Spasticity levels were assessed using the Modified Ashworth Scale (MAS) before and after each session, and also one week before (pre-intervention) and one week after (post-intervention) the program (Figure 1). The sessions were conducted at a community exercise facility in Denton, TX, USA.

The RE sessions began with a 10 min warm-up, involving assisted dynamic stretching targeting the neck, shoulders, elbows, and wrists. Grip-aid gloves were used to assist the participant with securely holding equipment, as spasticity in the hands limited voluntary control and grip strength. These gloves provided the necessary support to maintain a stable hold during exercises, promoting greater independence and safety in movement. The main exercise phase focused on the upper body, shoulder, and trunk muscles. The routine included five exercises: inclined dumbbell press, tubing band pull, shoulder press machine, seated tubing band “good morning”, and medicine ball trunk rotation. Each exercise was performed for three sets of 10–12 repetitions, with a 45 s rest between sets and a 2-min break between exercises. The session concluded with a 10 min cool-down, incorporating static assisted stretching and breathing exercises to aid in recovery.

The BE sessions began with a 10 min warm-up, featuring assisted dynamic stretching of the shoulders, arms, chest, and back. The main phase involved alternating moderate-and vigorous-intensity intervals. The participant completed four sets of 20 s moderate-intensity intervals on the hand cycle bike, followed by 40 s of rest. This targeted the deltoid, biceps, triceps, and pectoral muscles. Afterward, three sets of 10 s high-intensity intervals with 50 s rest periods were performed to focus on endurance and strength. A final set of four 20 s moderate-intensity intervals was completed before a 10 min cool-down, including static stretching and breathing exercises.

AE sessions were designed to improve upper body strength and flexibility while reducing the effects of spasticity. The participant wore a lifeguard jacket and ankle weights for safety and stability. Without the ankle weights, the participant’s lower body tended to float due to limited motor control, making it difficult to maintain a stable and upright position during activities. A 10 min warm-up outside the pool included assisted dynamic stretching of the upper body, shoulders, and trunk. The main phase consisted of three sets of 10–12 repetitions of resistance exercises in chest-deep water using aquatic barbells and dumbbells. These exercises, such as shoulder adduction, abduction, and trunk rotations, targeted the shoulders, chest, upper back, and core muscles. The session concluded with a 10 min cool-down involving static stretching and breathing exercises for flexibility and recovery.

### 2.3. Measurement

Spasticity was assessed using the MAS, a widely recognized and validated method for evaluating muscle spasticity [25]. The MAS measures the resistance of a muscle to passive stretching, indicating the level of spasticity. It uses a scoring system from 0 to 4 to quantify muscle stiffness: 0 (no increase in muscle tone), 1 (slight increase at the end of the range of motion), 1+ (slight increase with a catch or minimal resistance less than half of the range), 2 (marked increase but easy movement), 3 (considerable increase with difficult passive movement), and 4 (rigid in flexion or extension, severe spasticity). The MAS scores were considered ordinal, and a value of 1.5 was assigned to ratings of 1+ to maintain equal intervals [27,28].

In this study, spasticity was measured in specific muscle groups of the affected extremity, including the elbow flexor, elbow extensor, shoulder flexor, shoulder extensor, shoulder abductor, and shoulder adductor. Two certified physical therapists conducted all measurements to ensure accuracy and consistency.

Spasticity was evaluated at two key time points: pre-session and immediately following the exercise session. Pre-intervention measurements established a baseline, while post-intervention measurements allowed for a comparison of changes in spasticity levels.

### 2.4. Data Analysis

Data analysis for this study was conducted using SPSS version 23. To evaluate the intervention’s impact, paired samples *t*-tests were performed to compare pre- and post-intervention MAS scores within each exercise modality (RE, BE, and AE). This determined if there were statistically significant changes in spasticity between pre- and post-intervention within each modality. The significance level was set at *p* < 0.05.

Additionally, a one-way ANOVA was used to analyze the effect of each exercise modality on the mean difference in MAS scores from pre- to post-intervention. This test compared the effectiveness of the three modalities (RE, BE, and AE) in reducing muscle spasticity over the 9-week program. A Bonferroni post hoc test followed the ANOVA to pinpoint specific differences in mean change scores between the modalities, revealing which exercise modality produced the most significant reduction in spasticity.

Weekly spasticity measurements were also tracked and analyzed throughout the 9-week intervention. The mean weekly changes in MAS scores for each muscle group were calculated and aggregated to identify trends over time. This analysis offered valuable insights into whether spasticity decreased consistently, remained stable, or fluctuated throughout the program.

## 3. Results

This study assessed changes in spasticity using the MAS before and after each session throughout the 9-week intervention. Table 1 presents the change in mean MAS score both overall and for each specific modality. The results showed a significant decrease in the total MAS score, from 2.76 to 2.33 (*p* < 0.05). Each modality (RE, BE, AE) demonstrated statistically significant reductions in spasticity, with the AE modality showing the largest decrease (2.81 to 2.10), and all modalities exhibiting a high effect size (d > 0.8)

A one-way independent-sample ANOVA was conducted to investigate the impact of exercise modality—RE, BE, and AE—on the reduction in MAS scores. The analysis revealed a significant effect of exercise modality on MAS score reduction: F(2,15) = 27.20, *p* < 0.001, ηp^2^ = 0.78. This large effect size suggests that the type of exercise performed had a substantial impact on the reduction in muscle spasticity.

Post hoc comparisons using the Bonferroni test further indicated that participation in AE (M = 0.71, SD = 0.14) resulted a significantly greater reduction in MAS scores compared to BE (M = 0.39, SD = 0.09) and RE (M = 0.18, SD = 0.13). Additionally, the reduction in MAS scores for BE was significantly greater than for RE (Table 2). These results suggest that AE was the most effective modality for reducing muscle spasticity, followed by BE, with RE being the least effective.

**Figure 2:**
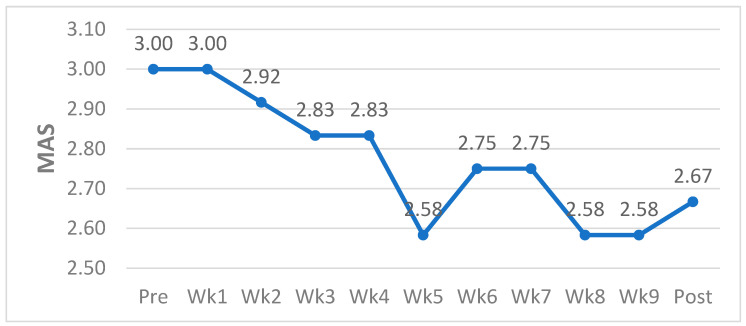
Overall changes in spasticity.

To assess overall changes in spasticity, including measurements taken one week prior to the intervention (PRE-intervention) and one week after the intervention (POST-intervention), weekly MAS scores were tracked and analyzed (Figure 2). The overall spasticity started with a mean MAS score of 3.00 at the PRE-intervention assessment and decreased to 2.67 at the POST-intervention assessment, showing a total reduction of 0.33. The lowest MAS score recorded during the intervention period was 2.58, indicating a consistent decrease in spasticity across the 9-week program. This trend suggests that the intervention was effective in gradually reducing muscle spasticity over time.

## 4. Discussion

This single case study examined the effects of three exercise interventions—weight-resistance exercise, hand cycle biking, and aquatic exercise—on upper extremity spasticity in an adult with quadriplegic CP. Spasticity, a hallmark of CP, often leads to pain, joint deformities, and reduced mobility, which in turn diminish functional independence and overall well-being [10,29]. Given the prevalence of upper extremity involvement in CP, interventions targeting spasticity in this region are crucial for improving both physical function and quality of life [30,31]. While research has demonstrated the benefits of exercise for improving general fitness and motor function in individuals with CP, the specific impact on spasticity remains less explored. This study contributes to the growing body of literature by focusing on spasticity as a primary outcome and comparing the effects of different exercise modalities.

The results of this study suggest that weight-resistance exercise has the potential to reduce spasticity in the upper extremities, although the improvements were modest (0.18). The participant exhibited a slight reduction in MAS scores following the resistance exercise sessions, indicating a decrease in muscle tone. These findings align with previous studies suggesting that resistance exercise may improve muscle strength and function without exacerbating spasticity in individuals with CP [15,16]. The routine, which targeted the upper body and trunk muscles, could have contributed to greater muscle control, leading to reduced involuntary muscle contractions. However, it is important to note that while resistance exercise showed some positive effects, spasticity did not significantly improve over the course of the intervention, suggesting that resistance exercise alone may not be sufficient to substantially reduce spasticity in severe cases of CP. Further research could explore whether varying the intensity or frequency of resistance exercise leads to greater improvements in muscle tone.

Hand cycle biking demonstrated more noticeable effects on spasticity, with the participant showing moderate reductions in MAS scores post-intervention (0.39). The use of hand cycling engages multiple upper body muscle groups, including the shoulders, chest, and arms, and may help maintain joint mobility and reduce muscle stiffness. Studies have highlighted the benefits of cycling exercises for improving both cardiorespiratory fitness and joint mobility in individuals with neuromuscular conditions [17,22]. This study adds to this evidence by showing that hand cycle biking can also lead to reductions in spasticity, particularly in the upper extremities. It is plausible that the repetitive, controlled movement of the hand cycle bike helped to alleviate muscle tightness and promote more relaxed muscle states [17]. The ability of the exercise to target spasticity without exacerbating it further underscores its potential as an effective and practical modality for individuals with quadriplegic CP.

Among the three modalities, aquatic exercise yielded the most significant reduction in spasticity (0.71), with the participant showing a consistent decline in MAS scores over the intervention period. The buoyant properties of water provide a supportive environment that reduces the effects of gravity, allowing individuals with CP to perform movements with reduced resistance and joint strain [23]. This could explain why the participant experienced the greatest improvement in muscle tone following aquatic exercise sessions. Water-based exercises not only promote muscle relaxation, but also engage multiple muscle groups, enhancing both strength and flexibility [24]. These findings are consistent with the existing literature highlighting the benefits of aquatic therapy in reducing spasticity and improving motor function in individuals with CP [14,18]. Aquatic exercise, therefore, presents a particularly effective strategy for managing spasticity in adults with quadriplegic CP, particularly when integrated into a comprehensive rehabilitation program.

The findings from this study demonstrate that different exercise modalities exert varying degrees of influence on spasticity. Aquatic exercise emerged as the most effective intervention, followed by hand cycle biking, while resistance exercise had the least impact. These results suggest that exercises incorporating dynamic, rhythmic movements—such as those seen in cycling and aquatic exercise—are more effective in reducing spasticity compared to static, strength-based exercises [32,33]. This supports the theory that spasticity may respond better to activities that promote flexibility and joint mobility rather than exercises that solely focus on muscle strengthening [17,20]. The lack of significant improvements in spasticity following resistance exercise may indicate that although strength training is beneficial for muscle function, its impact on reducing involuntary muscle contractions is limited [34]. The variability in outcomes highlights the need for individualized exercise programs tailored to the unique needs and physical capacities of individuals with CP.

Several limitations of this study should be considered. First, the focus on a single participant limits the generalizability of the findings. Additionally, the short intervention duration of nine weeks may not have been long enough to observe sustained changes in spasticity, particularly given the chronic nature of CP. Furthermore, although the exercise modalities were randomized to reduce bias, the session order may have influenced outcomes. Potential cumulative or interaction effects between sessions cannot be entirely ruled out, and the independent effectiveness of each modality may be further examined in future research.

Future research should explore the long-term effects of exercise interventions on spasticity in individuals with CP, particularly using extended intervention periods to better assess sustained outcomes. Studies with larger and more diverse samples are needed to enhance generalizability and strengthen the reliability of findings. Additionally, investigating the independent effectiveness of each exercise modality in isolation, as well as in different sequences, may help clarify their specific contributions. Further research may also consider the potential benefits of combining exercise with other therapeutic approaches, such as pharmacological treatments or botulinum toxin injections, to optimize spasticity management.

## 5. Conclusions

This study provides valuable insights into the effects of weight-resistance exercise, hand cycle biking, and aquatic exercise on upper extremity spasticity in an adult with quadriplegic CP. While all three interventions effectively reduced spasticity, aquatic exercise was the most impactful, followed by hand cycle biking, with resistance exercise showing the least effect. These findings highlight the importance of incorporating dynamic, rhythmic exercises, especially aquatic exercises, into rehabilitation programs for individuals with severe spasticity. Although resistance training is prioritized for strength and hypertrophy, this study suggests that it is less effective in reducing spasticity compared to other modalities. Practitioners working with individuals with severe spasticity should consider aquatic exercise as a primary intervention. Combining multiple modalities in a structured program may offer synergistic benefits, improving both muscle strength and spasticity management for individuals with CP.

## Figures and Tables

**Table 1:** Mean change in spasticity between different exercise modalities.

Modality	Pre-Test M (SD)	Post-Test M (SD)	t (df)	*p* Value	*d*
Total	2.76 (0.17)	2.33 (0.31)	7.16 (1, 17)	0.01 *	0.75
RE	2.72 (0.20)	2.54 (0.23)	3.31 (1, 5)	0.02 *	0.68
BE	2.75 (0.17)	2.36 (0.24)	11.06 (1, 5)	0.01 *	0.96
AQ	2.81 (0.16)	2.10 (0.29)	11.83 (1, 5)	0.01 *	0.96

Abbreviations: M, mean; SD, standard deviation; t (df) = t value (degree of freedom); *d =* Cohen’s d; RE, resistance exercise; BE, hand cycle bike exercise; AQ, aquatic exercise; * = *p* < 0.05.

**Table 2:** One-way ANOVA results.

Modality	Mean	SD	F	*p* Value	ηp^2^
RE	0.18	0.13	27.20	0.001 *	0.784
BE	0.39	0.09			
AQ	0.71	0.15			
Difference					
Modality	Difference	*p* value	95% confidence interval (LB-UB)		
AQ-RE	0.53	0.001	0.33		0.72
AQ-BE	0.32	0.001	0.12		0.51
BE-RE	0.20	0.034	0.01		0.40

Abbreviations: SD, standard deviation; ηp^2^, partial eta square; LB-UB, lower bound and upper bound; RE, resistance exercise; BE, hand cycle bike exercise; AQ, aquatic exercise; * = *p* < 0.05.

## Data Availability

Data are available on request due to restrictions.
